# Metabolic Functions of G Protein-Coupled Receptors in Hepatocytes—Potential Applications for Diabetes and NAFLD

**DOI:** 10.3390/biom10101445

**Published:** 2020-10-15

**Authors:** Takefumi Kimura, Sai P. Pydi, Jonathan Pham, Naoki Tanaka

**Affiliations:** 1 Molecular Signaling Section, Laboratory of Bioorganic Chemistry, National Institute of Diabetes and Digestive and Kidney Diseases, National Institutes of Health, Bethesda, MD 20894, USA; saiprasad.pydi@nih.gov (S.P.P.); jpham196@gmail.com (J.P.); 2 Department of Internal Medicine, Division of Gastroenterology, Shinshu University School of Medicine, Matsumoto 390-8621, Japan; 3 Department of Metabolic Regulation, Shinshu University School of Medicine, Matsumoto 390-8621, Japan; naopi@shinshu-u.ac.jp; 4 Research Center for Social Systems, Shinshu University, Matsumoto 390-8621, Japan

**Keywords:** G protein-coupled receptor, GPCR, hepatocyte, liver, metabolism, diabetes, NAFLD, NASH

## Abstract

G protein-coupled receptors (GPCRs) are cell surface receptors that mediate the function of extracellular ligands. Understanding how GPCRs work at the molecular level has important therapeutic implications, as 30–40% of the drugs currently in clinical use mediate therapeutic effects by acting on GPCRs. Like many other cell types, liver function is regulated by GPCRs. More than 50 different GPCRs are predicted to be expressed in the mouse liver. However, knowledge of how GPCRs regulate liver metabolism is limited. A better understanding of the metabolic role of GPCRs in hepatocytes, the dominant constituent cells of the liver, could lead to the development of novel drugs that are clinically useful for the treatment of various metabolic diseases, including type 2 diabetes, nonalcoholic fatty liver disease (NAFLD) and nonalcoholic steatohepatitis (NASH). In this review, we describe the functions of multiple GPCRs expressed in hepatocytes and their role in metabolic processes.

## 1. Introduction

The liver is the crucial orchestrator of metabolism. In humans, approximately 11,000 genes are actively transcribed in hepatocytes, and many of them are related to the metabolic functions of the liver [1]. The liver mediates numerous physiological functions, including protein synthesis, macronutrient metabolism, cholesterol and lipid homeostasis, endocrine control of growth signaling pathways, and drug detoxification [2,3,4,5,6]. Metabolic hormones, nutrients, and sympathetic and parasympathetic neural signals tightly regulate liver function [7,8]. Alterations in these signaling pathways lead to the dysfunction of liver metabolism and can cause insulin resistance, type 2 diabetes (T2D), nonalcoholic fatty liver disease (NAFLD), nonalcoholic steatohepatitis (NASH), and cancer [7,8,9]. The liver consists of hepatocytes, biliary epithelial cells, stellate cells, smooth muscle cells, vascular endothelial cells, various immune cells, and sinusoidal endothelial cells [10,11,12]. Each of these cell types possesses a unique function and collectively regulates liver function at multiple levels. Hepatocytes are the primary cell type present in the liver and account for 80% of the liver cell population [12].

**Figure 1:**
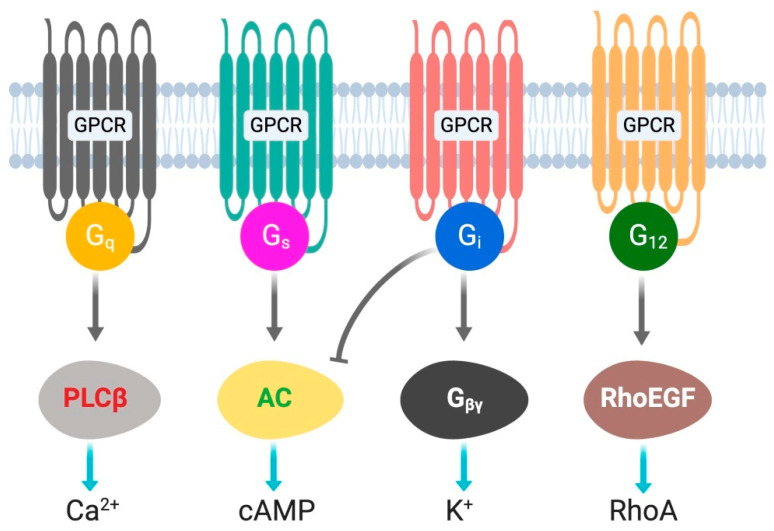
G-proteins’ classification and downstream signals.

G protein-coupled receptors (GPCRs) are cell surface receptors and mediate the function of a vast number of extracellular ligands (neurotransmitters, hormones, etc.) [13,14]. Ligands-coupled GPCRs recognize and activate heterotrimeric G proteins, which comprise Gα, Gβ, and Gγ. Gα determines the functions of a G protein through the exchange of guanosine diphosphate (GDP) with guanosine triphosphate (GTP) [15]. The Gβ and Gγ subunits are separately synthesized and form a complex to function as a single functional unit [16]. Both the Gα and Gβγ subunits activate signaling molecules and trigger cellular responses. Based on their α subunit, G proteins are classified into four families: G_s_, G_i_, G_q_, and G_12/13_ (Figure 1), where s and i represent stimulation and inhibition, respectively. Receptor-activated G proteins stimulate various downstream signaling pathways; for example, G_s_ and G_i_ proteins control adenylyl cyclase activity, G_q_ activates phospholipase Cβ, and G_12/13_ stimulates guanine nucleotide exchange factors for small GTPases of the Rho family [17,18]. The human genome contains about 800 different GPCR genes, which represent 3–4% of all human genes. Importantly, 30–40% of the drugs currently used in the clinic mediate their therapeutic effects via acting on GPCRs [13]. Understanding how GPCRs work at the molecular level is, therefore, of considerable therapeutic relevance.

Similar to many other cell types, hepatocyte function is regulated by GPCRs [19,20,21,22]. More than 50 different GPCRs are predicted to be expressed by mouse liver [23]. However, our knowledge about how GPCRs regulate liver function remains incomplete. In this review, we discuss the function of various GPCRs expressed in hepatocytes and their roles in various metabolic processes. The receptors mentioned in this review were selected with reference to the data quantifying the transcriptional levels of 353 GPCRs in mouse tissues described by Regard et al. [23] and GPCR-related paper data in hepatocytes reported in PubMed. For the classification of α subunit, we referred to the IUPHAR/BPS Guide to Pharmacology [24], including those belonging to multiple families. We focused mainly on the critical GPCR functions in hepatocyte and their role in liver metabolism.

## 2. Role of Gs-Coupled GPCRs in Liver Metabolism

### 2.1. Glucagon Receptor

**Figure 2:**
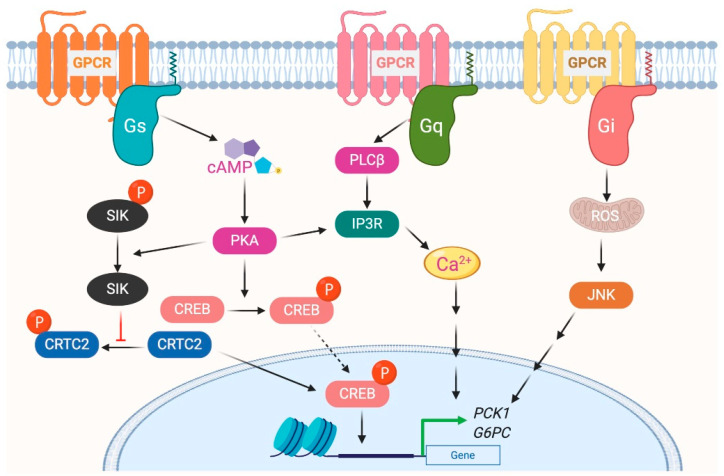
G protein-coupled receptors-mediated glycogenesis in hepatocytes.

The glucagon receptor (GCGR) is abundantly expressed by hepatocytes and is the most extensively studied Gs-coupled GPCR in the liver [25]. GCGR is activated by the 29 amino acid polypeptide glucagon, which is secreted by α cells of the endocrine pancreas, in particular during the fasted state or exercise [26]. The primary function of glucagon is to prevent hypoglycemia during fasting by increasing hepatic glucose production (HGP) from hepatocytes by stimulating both glycogenolysis and gluconeogenesis [27,28]. Activation of GCGR in hepatocytes results in increased intracellular cyclic adenosine monophosphate (cAMP) levels, followed by activation of protein kinase A (PKA) and cAMP response element-binding (CREB) protein (Figure 2). Phosphorylated CREB (the active form of CREB) binds to CRE and increases the expression of essential gluconeogenic genes, including glucose 6-phosphatase (*G6pc*) and phosphoenolpyruvate carboxykinase (*Pck1*) [29]. The activated GCGR also stimulates Ca^2+^ release from the endoplasmic reticulum (ER) and activates calcium/calmodulin-dependent protein kinase II (CAMKII). This results in the translocation of FOXO1 to the nucleus that promotes the transcription of gluconeogenic genes [30]. In addition to transcriptional regulation of genes involved in glucose metabolism, GCGR activation also modulates the activity of enzymes involved in glucose metabolism via PKA-dependent phosphorylation. PKA phosphorylates phospho-fructokinase 2 (PFK-2), fructose 2,6-bisphosphatase (FBPase2), pyruvate kinase, and phosphorylase kinase. Phosphorylation of these enzymes increases HGP by increasing gluconeogenesis and glycogenolysis or by inhibiting glycolysis [25,31].

Kim et al. examined the effects of GCGR agonists on insulin action and glucose homeostasis [32]. The acute GCGR agonist immediately induced a hyperglycemic state, followed by improved glucose tolerance and glucose-stimulated insulin secretion. Furthermore, acute GCGR agonists improved insulin tolerance in a dose-dependent manner in both lean and obese mice. Phosphorylation of hepatic AKT at Ser473 was increased in mice treated with insulin and GCGR agonists. This effect was replicated in isolated primary mouse hepatocytes, increasing AKT kinase activity. These data suggest that hepatic GCGR activation improves glucose tolerance by enhancing the action of insulin. It has also been reported that hepatic GCGR stimulation reduces lipid content, drives glycogen flux, and improves mitochondrial turnover and function [33]. This glucagon signaling pointing mechanism has high potential applications in new drugs for NAFLD/NASH.

### 2.2. Thyrotropin-Releasing Hormone Receptor

Thyrotropin-releasing hormone (TRH) receptor (TRHR) is a Gq-coupled receptor; however, in hepatocytes, it was shown to signal through Gs [34]. TRHR is activated by TRH released from the hypothalamus. Activation of TRHR leads to increased cAMP levels and enhanced PKA activation, causing CREB activation and increased expression of gluconeogenic genes [34]. TRHR-activated PKA also phosphorylates salt-inducible kinase 2 (SIK2) and inhibits its activity [35,36]. Decreased SIK2-dependent phosphorylation of CRE2 and CREB-regulated transcription coactivator 2 (CRTC2) enables them to enter the nucleus and interact with CREB to promote the expression of the *G6pc* and *Pck1* genes [35,36], leading to enhanced HGP.

### 2.3. Beta-Adrenergic Receptor

Two catecholamines, epinephrine and norepinephrine released from the sympathetic nervous system, also regulate liver metabolism [37]. These catecholamines active Gs-coupled beta-adrenergic receptors (βARs), which are expressed in both rodent and human hepatocytes [38,39]. Two subtypes of βAR (β1AR and β2AR) are predominantly expressed in the liver, and their expression is known to increase with age [38]. βAR-mediated activation of adenylyl cyclase increases hepatic glucose output and liver lipid catabolism [40,41,42]. In rat hepatocytes, activation of βARs with nonselective agonist isoproterenol increases glycogen phosphorylase activity by many folds. Similar to GCGR, activation of β2AR in hepatocytes increases the expression of *Pck1*, *G6pc,* and l-pyruvate kinase (*pklr*) and decreases liver glycogen levels [42]. In a recent study, it was shown that activation of βAR signaling in hepatocytes increases the activity of hormone sensitive lipase (HSL) and adipose triglyceride lipase (ATGL) [42,43]. ATGL is the master regulator of triglyceride content in hepatocytes and it activates Sirtuin 1 (*Sirt1*), which can regulate expression of various lipid metabolic genes [44]. On the other hand, HSL in hepatocytes increases the activity of triglyceride hydrolase. HSL also plays a role in the hydrolysis of cholesterol esters [45]. Overall, activation of βARs in hepatocytes regulate HGP and lipid catabolism.

### 2.4. Prostaglandin E2 Receptor 4

The prostaglandin E2 receptor 4 (EP4) metabolic function in hepatocytes is mediated through Gs. EP4-associated protein (EPRAP) is a molecule downstream of EP4 that suppresses the inflammatory response of macrophages. [46]. In addition to macrophages, EPRAP is also expressed and detected in hepatocytes [47]. Higuchi et al. showed that *Eprap*-null mice fed on a high-fat sucrose diet showed significantly lower blood glucose levels compared to wild type (WT) mice when challenged with either glucose or pyruvate [47]. Furthermore, primary hepatocytes of *Eprap*-null mice showed reduced glucose production and reduced expression of *Pck1* and *G6pc* genes. Lentiviral-mediated suppression of hepatic EPRAP also reduced glucose levels and glucogenic gene expression in *db/db* mice. In summary, the authors concluded that EPRAP regulates hypoglycemia in diabetic mice by controlling hepatic gluconeogenesis. As stated by the authors, liver-specific deletion of *Eprap* is necessary for further understanding the role of hepatic EPRAP in glucose metabolism.

### 2.5. Sphingosine-1-Phosphate Receptor 2

Recently, the role of sphingosine-1-phosphate receptor 2 (S1PR2) in bile acid-mediated hepatic lipid metabolism was clarified [48]. S1PR2-mediated signaling includes Gs, Gq, and G12/13 mechanisms. In rodent primary hepatocytes, conjugated bile acids activated S1PR2, leading to the downstream activation of extracellular signal-regulated kinase 1/2 (ERK1/2) and AKT signaling pathways. Bile acid-mediated activation of ERK1/2 and AKT signaling pathways involves the regulation of intrahepatic glucose and lipid metabolism [49]. In primary rat hepatocytes, both insulin and bile acids similarly activated glycogen synthase activity. Infusion of taurocholate (TCA) into the chronic cholestine fistula rat activated the AKT and ERK1/2 signaling and glycogen synthase activity [50]. In addition, TCA reduced the expression of liver gluconeogenic genes, *Pck1*, and *G6pc* expression, and increased the expression of short heterodimeric partner (*Shp*) mRNA significantly [51]. These results suggest that TCA has an insulin-like activity to regulate hepatic glucose metabolism both in vitro and in vivo. Additionally, it has been reported that *S1pr2*-null mice fed a high-fat diet develop fatty liver more rapidly than wild-type mice, suggesting that S1PR2 is a vital regulator of hepatic lipid metabolism [52]. Infusion of TCA into the chronic bile fistula rat or overexpression of the gene encoding S1PR2 in mouse hepatocytes upregulated hepatic sphingosine kinase 2 (SphK2). Interestingly, the major genes encoding nuclear receptors/enzymes responsible for nutrient metabolism were downregulated in the livers of *S1pr2*-null and *Sphk2*-null mice. Consistently, overexpression of the gene encoding S1PR2 in primary mouse hepatocytes raised the mRNA levels of key genes associated with nutrient metabolism.

## 3. Role of Gi-Coupled GPCRs in Liver Metabolism

GPCRs that preferentially couple to Gi proteins inhibit the function of adenylyl cyclase and thereby decrease intracellular cAMP. In a recent study, to understand the role of hepatic Gi signaling on whole-body glucose metabolism, Dr. Wess’s group employed a chemo-genetic approach where a designer Gi-coupled receptor was selectively expressed in mouse hepatocytes (Hep-GiD). This designer receptor can be selectively activated by clozapine-N-oxide (CNO), an otherwise chemically inert compound [20]. As activation of the Gs-coupled GCGR increases HGP, it was expected that activation of Gi in the liver would have opposite effects. Interestingly, activation of Gi signaling in hepatocytes also increased HGP by activating c-Jun N-terminal kinases (JNK) signaling [20]. Activation of Gi signaling in mouse liver impaired glucose tolerance and stimulated gluconeogenesis and glycogenolysis. Both in vivo and in vitro studies showed that enhanced Gi signaling in hepatocytes led to a JNK-dependent increase in the expression of *G6pc* (Figure 2). Similar results were obtained with the human hepatocytes. Activation of Gi signaling in the liver did not affect hepatic insulin signaling [20].

### 3.1. Cannabinoid Receptor 1

To validate the findings obtained in the previous section with Hep-GiD mice, the authors targeted the cannabinoid receptor 1 (CB1), an endogenous Gi-coupled receptor expressed in hepatocytes. Acute activation of CB1 with anandamide in mice severely impaired glucose tolerance and increased glucose release from hepatocytes via enhanced reactive oxygen species (ROS) production, resulting in JNK activation and increased HGP [20]. A separate study by Dr. Kunos’s lab showed that activation of CB1 increased glycogen phosphorylase activity by 70%, suggesting that enhanced glycogenolysis is the primary source of the increased HGP [53]. CB1 also inhibited insulin signaling in hepatocytes by activating the Bip/PERK/eIF2*α* ER stress pathway [54,55]. Furthermore, another study showed that peripheral CB1 blockade in obese mice improved glycemic in control mice via the hepatic Sirt1/mTORC2/Akt pathway and increased fatty acid oxidation via LKB1/AMPK signaling [56].

### 3.2. A1 and A3 Adenosine Receptor

Another group of Gi-coupled receptors expressed in hepatocytes are the A1 and A3 adenosine receptors. Treatment of hepatocytes with the A1 receptor-selective agonist 2-chloro-N6-cyclopentyladenosine (CCPA) stimulates gluconeogenesis and glycogenolysis [57]. This receptor also regulates lipid metabolism in hepatocytes by increasing triglyceride synthesis by enhancing the expression levels of sterol regulatory element-binding protein 1 (SREBP-1) and peroxisome proliferator-activated receptor-gamma (PPARg), key regulators of genes involved in lipogenesis (*Fas*, *Acc*, *Acl*, stearoyl-CoA desaturase-1, and l-α-glycerophosphate acyltransferase) [58]. Similar to the A1 receptor, activation of the A3 receptor in hepatocytes increases HGP by enhancing gluconeogenesis and glycogenolysis [59,60].

### 3.3. GPR109A

GPR109A (HCA2, hydroxycarboxylic acid receptor 2) is a Gi-coupled receptor, and activation of GPR109A in hepatocytes by niacin decreases intracellular cAMP levels. Agonist stimulation of GPR109A in mouse hepatocytes decreases plasma high-density lipoprotein (HDL)-cholesterol by reducing the expression of the adenosine triphosphate (ATP) binding cassette transporter A1 (*Abca1*) and reduces the efflux of cholesterol to HDL [61,62]. Activation of either Gs or Gi signaling pathways in hepatocytes increases HGP. However, both signaling molecules use different signaling pathways. Interestingly, the A1 adenosine receptor and GPR109A Gi-coupled receptors also regulate lipid metabolism.

### 3.4. C-C Chemokine Receptor Types 2 and 5

The expression of Gi-coupled receptors C-C chemokine receptor type 2 (CCR2), and C-C chemokine receptor type 5 (CCR5) is increased in the livers of patients with alcoholic liver disease (ALD) [63]. Ambate et al. tested whether inhibition of CCR2/5-related signaling could inhibit ALD progression [63]. The authors used cenicriviroc (CVC), a dual inhibitor of CCR2/5, to inhibit CCR2/5 signaling in a mouse model of ALD, which reduced liver damage and steatosis. In vitro, CVC reduced expression of fatty acid synthase (*Fasn*) and adipose differentiation-related protein (*Adrp*) and increased the expression of acyl-coenzyme A oxidase 1 (*Acox-1*), proliferator-activated receptor gamma coactivator alpha (*Pgc1*α), and uncoupling protein two. These data suggested possible mechanisms for attenuated hepatocyte steatosis. Collectively, this study showed an association between alcohol-induced steatohepatitis, liver damage, and CCR2/5, indicating that inhibition of CCR2 and CCR5 may be an effective treatment for ALD.

### 3.5. Purinergic Receptor P2Y, G Protein-Coupled, 13

In cultured hepatocytes, activation of the Gi-coupled receptor, purinergic receptor P2Y13 (P2Y13), was reported to be essential for high-density lipoprotein (HDL) uptake [64]. The primary atheroprotective function of HDL is to promote “reverse cholesterol transport” (RCT). HDL mediates the efflux and transport of cholesterol from peripheral cells and subsequent metabolism and traffic to the liver for biliary excretion. Recently, P2Y13 has been shown to be a new target for regulating RCT [65]. This is a very relevant finding for the link between the liver and atherosclerosis, and the therapeutic application of these findings is expected.

### 3.6. Smoothened Receptor

The hedgehog (Hh) signaling pathway regulates the fate of hepatic progenitor cells and liver development [66]. Components of Hh signaling include the Gi- or G12/13-coupled receptor, smoothened (SMO) [67]. Activation of Hh has been observed in patients with NAFLD, but the function of hepatic Hh signaling in the pathogenesis of NAFLD was previously unknown. A recent publication examined the effects and mechanisms of hepatic Hh signaling in high fat diet (HFD)-induced NAFLD using genetic and pharmacological approaches [67]. Treatment of HFD-fed WT mice with Smo inhibitors (GDC-0449 and LED225) significantly reduced the number of activated macrophages and expression of pro-inflammatory cytokines. The SMO inhibitors were noted to have variable effects on hepatic fat accumulation. Hepatocyte-specific deletion of *Smo* reduced macrophage activation and inhibited the expression of pro-inflammatory cytokines but did not significantly alter fat accumulation in the liver. This paper concluded that hepatocyte Hh signaling played an important role in the progression of NAFLD by promoting hepatic inflammation via osteopontin-mediated macrophage activation, rather than a direct effect on the fat deposition. Selective inhibition of hepatocyte Hh signaling in patients with NAFLD might inhibit the progression from simple fatty liver to NASH.

## 4. Role of Gq-Coupled GPCRs in Liver Metabolism

Stimulation of Gq signaling leads to the activation of phospholipase Cβ, followed by an increase in intracellular levels of inositol trisphosphate, diacylglycerol, and calcium (Figure 1). Further downstream effectors of the signaling pathway are dependent on the receptor and cell type. To understand the role of hepatic Gq signaling on glucose homeostasis, Dr. Wess’s group expressed a Gq coupled, CNO-sensitive designer receptor selectively in mouse hepatocytes (Hep-GqD) [21]. Acute activation of this receptor with CNO increased blood glucose levels in a concentration-dependent manner. Hep-GqD mice showed impaired glucose and pyruvate tolerance. Impairments were predominately due to an increase in HGP [20]. Mechanistic studies confirmed an increase in the expression of various genes involved in gluconeogenesis and glycogenolysis. In particular, mRNA levels of *Pck1*, a rate-limiting enzyme of gluconeogenesis, were significantly increased (Figure 2). Similarly, the expression levels of genes involved in fatty acid syntheses such as *Acyl*, *Acc*, and *Fas* were also upregulated [20]. These data indicate that Gq signaling in hepatocytes plays a crucial role in glucose and lipid handling.

### 4.1. Vasopressin and Oxytocin Receptor

Hepatocytes express various Gq-coupled receptors, including arginine vasopressin receptor 1A (AVPR1A) and oxytocin (OT) [68,69,70,71]. The AVPR1A (endogenous agonist: arginine-vasopressin, AVP) is expressed predominantly in mammalian hepatocytes. Activation of AVPR1A in hepatocytes initiates glycogenolysis by phosphorylating glycogen phosphorylase (*Pygl*). In rat hepatocytes, activation of AVPR1A leads to increased glycogenolysis and gluconeogenesis [72,73]. Acute injection of AVP into experimental animals increases blood glucose levels, indicating that AVPR1A stimulates HGP [74,75,76].

Chronic activation of oxytocin receptors in lean mice via long-term treatment with oxytocin increased the expression of gluconeogenic genes such as *G6pc*, *Fbp1*, and *Pck1*. These mice also showed decreased liver glycogen levels and increased expression of hepatic *Pygl* [70,71,77]. In agreement with the in vivo data, the treatment of rat hepatocytes with oxytocin increased glycogen phosphorylase activity and decreased glycogen synthase activity [71]. Collectively, these data suggest that oxytocin increases net hepatic glucose oxidation.

### 4.2. Purinergic Receptor

Purinergic receptor P2Y receptors are activated by various extracellular nucleotides and nucleotide sugars. The presence of the Gq-coupled P2Y1, P2Y2, P2Y4, and P2Y6 receptors have been detected in hepatocytes [78]. Studies with human hepatocytes suggest that P2Y2 and P2Y4 modulate glycogen metabolism by reducing glycogen synthesis and promoting glycogenolysis [78,79]. Studies on rat hepatocytes showed that activation of P2Y1 stimulates glycogen phosphorylase [78].

### 4.3. Angiotensin II Type I Receptor

The Gq-coupled angiotensin II type I receptor (AT1R) is known to regulate cardiovascular function [80]. However, AT1R stimulation can also lead to hepatic stellate cell activation and fibrosis via phosphorylation of janus kinase 2 (JAK2) as well as activation of RhoA and Rho-associated kinase 1 (ROCK1). In primary rat hepatocytes, the angiotensin II analog [Sar1, Ile4, Ile8]-angiotensin II (SII Ang II) showed positive effects on insulin receptor signaling and glucose metabolism [81]. Long-term pretreatment with SII Ang II enhanced insulin-stimulated glycogen synthesis and promoted insulin-stimulated phosphorylation of Akt and GSK3a/b, but not of FOXO1 [81]. Furthermore, the effects of SII Ang II on insulin-stimulated signaling and glycogen synthesis were dependent on the activity of Src and Gaq, since inhibitors of these proteins abolished the beneficial metabolic effects of SII Ang II [81].

### 4.4. Free Fatty Acid Receptor 4 and GPR40

Free fatty acid receptor 4 (FFA4, GPR120) is a known Gq-coupled receptor and has been suggested as a target of omega-3 polyunsaturated fatty acids (n-3 PUFAs) [82]. Kang et al. evaluated the functional role of FFA4 in hepatic steatosis using an in vitro model of liver X receptor (LXR)-mediated hepatic steatosis [82]. The expression of FFA4 in Hep3B and HepG2 human hepatocytes was first confirmed. T0901317 (a specific LXR activator) induced lipid accumulation and docosahexaenoic acid (DHA, one of the n-3 PUFAs) inhibited lipid accumulation. This DHA-induced inhibition was blocked by small interfering RNA (siRNA)-mediated FFA4 knockdown or treatment with FFA4 antagonist, AH7614. SREBP-1c (a vital transcription factor for adipogenesis) expression was induced by treatment with T0901317, and DHA inhibited SREBP-1c at the mRNA and protein levels. Furthermore, DHA inhibited T0901317-induced lipid accumulation in primary hepatocytes of WT mice, but not in primary hepatocytes of FFA4-deficient mice. These results suggest that FFA4, Gq/11 protein, and SREBP-1c suppression are sequentially involved in liver fat deposition.

On the other hand, there are conflicting views on the receptors that DHA operates on. The various bioactivities of n-3 PUFAs depend not only on the FFA4, but also on their agonistic effects on GPR40 (free fatty acid receptor 1, FFA1). On et al. evaluated whether the anti-lipogenic effects of DHA in hepatocytes were mediated through FFA4 or GPR40 using specific agonists (compound A for FFA4 and AMG-1638 for GPR40), hepatocytes from FFA4 knockout (KO) mice, and a GPR40 selective antagonist (GW1100) [83]. Compound A did not reduce SREBP-1 and FAS protein expression in hepatocytes exposed to T0901317 or high glucose with insulin. Furthermore, DHA reduced the expression of lipogenic enzymes in FFA4-null hepatocytes. On the contrary, AMG-1638 decreased SREBP-1 and stearoyl-coenzyme A denaturase 1 (SCD-1) protein levels. Moreover, GW1100, a GPR40 antagonist, reversed the anti-lipogenic effects of DHA. Based on these results, the authors concluded that DHA reduced the expression of SREBP-1-mediated lipid-metabolizing enzymes via GPR40 but not FFA4.

The protective effect of n-3 PUFAs on NAFLD patients has been reported [84,85]. Tanaka et al. tested the efficacy of eicosapentaenoic acid (EPA), one of the major members of the n-3 PUFAs, against NASH [85]. In this study, 23 patients with biopsy-proven NASH were treated with high-purity EPA (2700 mg/d) for 12 months, and their efficacy was assessed by biochemical parameters and liver histological examination. After 12 months, serum alanine aminotransferase levels, serum-free fatty acids, plasma soluble tumor necrosis factor receptor levels, and serum ferritin and thioredoxin levels, which may reflect hepatic oxidative stress, were significantly decreased. Seven of the 23 patients underwent post-treatment liver biopsies, and six patients had liver steatosis and fibrosis. Liver improvement in hepatocyte ballooning and lobular inflammation was observed. Although this was a small-scale study and the authors did not conclude that EPA is a specific treatment for NASH, this study suggested a positive impact of EPA on NASH pathogenesis due to its anti-inflammatory and antioxidant effects. There was a recent review of clinical trials conducted on n-3 PUFAs and NAFLD/NASH [84]. Very interestingly, 13 of 17 published studies reported that n-3 PUFAs supplementation decreased liver fat, liver enzymes, or markers of inflammation; four studies suggested a reduction in ballooning and two studies suggested a reduction in fibrosis. Results also indicated that DHA was more effective than EPA in the treatment of NAFLD. Caloric restriction and supplementation with n-3 PUFAs were additive in decreasing hepatic steatosis.

### 4.5. Prostaglandin E Receptor 1

Prostaglandin E receptor 1 (EP1) is also a Gq-coupled receptor, with a few metabolic studies on hepatocytes. Prostaglandin E has been reported both to stimulate glycogen-phosphorylase activity (glycogenolytic effect) and to inhibit the glucagon-stimulated glycogen-phosphorylase activity (anti-glycogenolytic effect) in rat hepatocytes [86]. This conflicting event has been reported to be caused via EP1 and prostaglandin E receptor 3 (EP3, Gi-coupled receptor), respectively [86]. However, there are still many unresolved aspects of these signal analyses. The functions of EP1/3 receptors in liver metabolism require further investigation.

### 4.6. Serotonin Receptor

In addition to the role as a neurotransmitter in the regulation of central nervous system function, serotonin (5-hydroxytryptamine, 5-HT) has metabolic functions in the periphery [87]. The action of 5-HT is dependent on the type of 5-HT receptors (types 1–7 5-HT receptors). Serotonin receptor 2A (5-HT2A) and serotonin receptor 2B (5-HT2B) are distributed in the liver [88]. Hepatic synthetic 5-HT acting on the serotonin receptor 2 (5-HT2) has been shown to regulate lipid-induced lipid over synthesis [88]. The combination of antagonists of both 5-HT synthesis and 5-HT2 significantly inhibited hepatic steatosis, inflammation, fibrosis, hyperglycemia, and dyslipidemia in diabetic mice. Thus, activation of the 5-HT system in hepatocytes plays a vital role in the induction of diabetes-related hepatic dysfunction and may be a potential therapeutic target.

## 5. Role of G_12/13_ Signaling in Liver Metabolism

### 5.1. Gα12

The G_12/13_ family comprises two members: Gα12 and Gα13. Kim et al. examined the regulatory role of G protein α12 (Gα12) on hepatic lipid metabolism and systemic energy expenditure [89]. Hepatic levels of Gα12 were increased by fasting in the livers of mice. The fasting-induced hepatic fat accumulation was significantly enhanced in *Gna12*-KO mice. The cDNA microarray analysis of *Gna12*-KO mouse livers demonstrated that the Gα12 signaling pathway regulates SIRT1 and PPARα, both of which are involved in mitochondrial respiration. SIRT1 was poorly induced in the livers of *Gna12*-KO mice during fasting, but Gα12 overexpression in lentivirus-mediated hepatocytes reversed this expression. Gα12 stabilized the SIRT1 protein by inducing transcription of ubiquitin-specific peptidase 22 (USP22) via increased HIF-1a. *Gna12*-KO mice fed a high-fat diet were more prone to diet-induced hepatic steatosis and obesity due to reduced energy expenditure. Furthermore, as an impactful result, liver biopsies of NAFLD patients had significantly reduced Gα12 levels. These results indicate that Gα12 regulates SIRT1-dependent mitochondrial respiration via HIF-1α-dependent USP22 induction, suggesting that Ga12 is an upstream molecule that contributes to the regulation of mitochondrial energy expenditure.

Kim et al. further argued that the adenosine receptor, which was also referred to above, is relevant to Gα12 signaling [89]. In embryonic fibroblasts, treatment of WT mice-derived cells with an agonist for adenosine significantly increased SIRT1 levels, whereas SIRT levels were lower in *Gα12*-KO mice-derived cells. Similar results were obtained using AML12 cells, stably expressing shRNA for Gα12. In addition, as evidence of hepatocyte involvement, primary hepatocytes exposed to adenosine receptor agonists showed a significant increase in SIRT1 and USP22. In addition, serum adenosine levels in mice were significantly increased by fasting. No change was observed in liver homogenates. Taken together, increased circulating adenosine concentrations, activation of adenosine receptors, and increased Gα12 may amplify SIRT1 induction and maintain systemic energy homeostasis.

### 5.2. Gα13

Kim et al. also explored differences in the number of proteins regulated by Gα13 in the liver using a proteomics-based approach [90]. Among the secreted proteins enriched in the liver, they identified inter-alpha-trypsin inhibitor heavy chain 1 (ITIH1) as a critical molecule associated with metabolic abnormalities. ITIH1 is a hyaluronan-binding protein that is predominantly expressed in hepatocytes under diabetic conditions. ITIH1 levels were reduced in patients with liver fibrosis, and circulating ITIH1 mRNA levels were increased in rats with D-galactosamine-induced liver injury. Hepatic Gα13 levels were reduced under diabetic conditions, in which ITIH1 is a major driver of cross talk between organs regulated by the liver. Hepatic Gα13 increased ITIH1 overexpression via O-linked β-N-acetylglucosamine transferase catalyzed O-GlcNAcylation. Overexpressed ITIH1 in the absence of hepatic Gα13 was secreted into the circulation and bound directly to adipose tissue and skeletal muscle hyaluronan, stabilizing their respective integrity and exacerbating peripheral insulin resistance. These results indicate that a liver Gα13-mediated signaling cascade is important for systemic glucose metabolism.

## 6. GPCR Signaling in Relation to Metformin, PPARγ Agonist, and Statins

The relationship between GPCR signaling and drugs already widely used for diabetes and dyslipidemia, such as metformin, PPARγ agonist, and statins, should also be discussed. Although these drugs are not direct GPCR stimulators, indirect effects on GPCR signaling can be occurring. 

We recently reported that activation of Gq-coupled receptors enhanced AMPK-mediated glucose uptake in muscle [91]. Whether there is a synergistic effect between hepatic AMPK activation by metformin and Gq receptor activation in the muscle (and other tissues, including liver) is a subject to be examined. Additionally, Paschoal et al. showed that GPR120 and PPARγ agonists functionally interact and markedly improve insulin resistance by using adipocytes and macrophages [92]. Also, chronic cholesterol depletion by statins in HEK cells has been shown to switch the serotonin 1A receptor (5-HT1A) endocytosis pathway from clathrin-mediated endocytosis to caveolin-mediated endocytosis [93]. Clarifying the interactions of these agents with GPCRs in the liver is an interesting topic for future research.

## 7. Closing Remarks

In addition to the receptors described Below (Table 1), many other GPCRs are present in hepatocytes, including many orphan receptors, but their potential roles in regulating hepatic glucose fluxes remain to be explored. An improved understanding of the metabolic functions of hepatocyte GPCRs may lead to the development of novel drugs that could prove clinically useful for the treatment of various metabolic disorders, including T2D, NAFLD, and NASH. 

## Figures and Tables

**Table 1:** G protein-coupled receptors involved in metabolic function of hepatocytes.

*Gene Symbol (Human)*	Receptor Name	Family	Transduction Mechanisms			
*GCGR*	Glucagon receptor (GCGR)	Glucagon receptors	Gs			
*TRHR*	Thyrotropin-releasing hormone receptor (TRH)	Thyrotropin-releasing hormone receptors	Gs	Gq		
*ADRB1*	β1-adrenoceptor (B1AR)	Adrenoceptors	Gs	Gi		
*ADRB2*	β2-adrenoceptor (B2AR)	Adrenoceptors	Gs	Gi		
*PTGER4*	Prostaglandin E receptor 4 (EP4)	Prostanoid receptors	Gs	Gi	Gq	
*S1PR2*	Sphingosine-1-phosphate receptor 2 (S1PR2)	Lysophospholipid (S1P) receptors	Gs	Gq	G_12/13_	
*CNR1*	Cannabinoid receptor 1 (CB1)	Cannabinoid receptors	Gi	Gs		
*ADORA1*	Adenosine A1 receptor (A1)	Adenosine receptors	Gi	Gq	Gs	G_12/13_
*ADORA3*	Adenosine A3 receptor (A3)	Adenosine receptors	Gi	G_12/13_		
*HCAR2*	Hydroxycarboxylic acid receptor 2 (HCA2, GPR109A)	Hydroxycarboxylic acid receptors	Gi			
*CCR2*	C-C chemokine receptor type 2 (CCR2)	Chemokine receptors	Gi			
*CCR5*	C-C chemokine receptor type 5 (CCR5)	Chemokine receptors	Gi			
*P2RY13*	Purinergic receptor P2Y13 (P2Y13)	P2Y receptors	Gi			
*SMO*	Smoothened receptor (SMO)	Class Frizzled GPCRs	Gi	G_12/13_		
*AVPR1A*	Arginine vasopressin receptor 1A (AVPR1A)	Vasopressin and oxytocin receptors	Gq			
*OXTR*	Oxytocin receptor (OT)	Vasopressin and oxytocin receptors	Gq	Gi		
*P2RY1*	Purinergic receptor P2Y1 (P2Y1)	P2Y receptors	Gq	Gi		
*P2RY2*	Purinergic receptor P2Y2 (P2Y2)	P2Y receptors	Gq	Gi	G_12/13_	
*P2RY4*	Purinergic receptor P2Y4 (P2Y3)	P2Y receptors	Gq			
*P2RY6*	Purinergic receptor P2Y6 (P2Y6)	P2Y receptors	Gq	G_12/13_		
*AGTR1*	Angiotensin II receptor type 1 (AT1)	Angiotensin receptors	Gq	Gi		
*FFAR4*	Free fatty acid receptor 4 (FFA4, CPR120)	Free fatty acid receptors	Gq			
*FFAR1*	Free fatty acid receptor 1 (FFA1, GPR40)	Free fatty acid receptors	Gq	Gs	Gi	
*PTGER1*	Prostaglandin E receptor 1 (EP1)	Prostanoid receptors	Gq	Gi		
*HTR2A*	5-hydroxytryptamine receptor 2A (5-HT2A)	5-Hydroxytryptamine receptors	Gq	Gi		
*HTR2B*	6-hydroxytryptamine receptor 2B (5-HT2B)	5-Hydroxytryptamine receptors	Gq			
